# Allele-specific methylation of *SSTR4* associated with aging and cognitive functions in patients with schizophrenia

**DOI:** 10.1371/journal.pone.0303038

**Published:** 2025-02-05

**Authors:** Rongrong Zhao, Huihui Shi, Yanqiu Wang, Tao Jiang, Yahui Xu

**Affiliations:** 1 The First Affiliated Hospital and College of Clinical Medicine of Henan University of Science and Technology, Luoyang, China; 2 The Second Affiliated Hospital of Xinxiang Medical University, Xinxiang, China; State University of New York Upstate Medical University, UNITED STATES OF AMERICA

## Abstract

The co-occurrence of alcohol use disorder (AUD) and schizophrenia is prevalent, with a rate of 33.7%. Previous research has suggested a genetic and epigenetic overlap between these two disorders. *SSTR4*, a member of the somatostatin receptor family, is implicated in various neurological and psychiatric conditions, including cognitive function, AUD, and schizophrenia. However, the role of genetic-epigenetic interactions involving *SSTR4* in patients with schizophrenia remains unexplored. In this study, we conducted an integration of publicly available datasets and identified allele-specific methylation patterns in SSTR4. Additionally, we pinpointed several genetic variants (rs17691954, rs11464356, rs3109190, and rs145879288) that influence the pace of aging and cognitive functions (rs705935) through their quantitative trait loci effects on CpG sites within *SSTR4*.

## Introduction

The comorbidity of alcohol use disorder (AUD) and schizophrenia is not only prevalent but also presents a complex challenge in psychiatric care, with significant implications for treatment and prognosis. The severity of this comorbidity, noting that AUDs are often comorbid with psychiatric disorders like schizophrenia, with a comorbidity rate of approximately 33.7% [1]. This high rate of comorbidity underscores the intertwined nature of these disorders and the need for integrated treatment approaches. A genome-wide analysis revealing genetic overlap between alcohol use behaviors, schizophrenia, and bipolar disorder further emphasizing the genetic and biological underpinnings of this comorbidity [2] and suggesting a shared genetic architecture with a complex relationship between these disorders at a molecular level. The clinical implications of this comorbidity are profound. For example, an alcohol-induced psychotic disorder (AIPD) study found a significant prevalence of AIPD among patients treated for alcohol use, with notable comorbidity with other psychiatric disorders, including schizophrenia [3]. This finding points to the intricate clinical presentation and the challenges in distinguishing and treating comorbid conditions. Moreover, the comorbidity of AUD and schizophrenia is not isolated to specific populations: sexual orientation minorities showed a higher rate of substance use disorders and mental health issues, including schizophrenia [4]. This indicates the need for culturally sensitive and diverse treatment strategies.

The abnormality of methylation in AUD and schizophrenia has been a focus of recent research, shedding light on the complex genetic and epigenetic interactions underlying these disorders. Goetjen et al. explored the methylation at the GABRA2 promoter in the context of AUD, revealing that methylation is associated with decreased GABRA2 gene expression, which extends to the GABRB1 gene, indicating a potential epigenetic mechanism in AUD [5] Similarly, Guidotti et al. investigated the DNA methylation/demethylation network in psychotic patients with a history of alcohol abuse, finding alterations in DNA-methyltransferase-1 (DNMT1) and other related enzymes, suggesting a complex interplay between alcohol abuse and epigenetic regulation in psychotic disorders [6]. McCunn et al. using magnetic resonance spectroscopy in a rat model of co-occurring AUD and schizophrenia revealed alterations in GABA and glutamine that may underlie alcohol drinking behavior, pointing towards potential epigenetic factors in the comorbidity of these disorders [7]. Furthermore, Longley et al. reviewed the role of DNA methylation in AUD, highlighting the rapid growth in studies investigating this epigenetic modification and its contribution to AUD, with a particular emphasis on the immune system’s involvement [8]. These studies collectively underscore the significance of methylation abnormalities in the etiology and progression of AUD and schizophrenia. They reveal a complex landscape where genetic predispositions, environmental factors, and epigenetic modifications interplay, contributing to the manifestation and comorbidity of these disorders. This growing body of research not only enhances our understanding of the molecular basis of AUD and schizophrenia but also opens avenues for potential therapeutic interventions targeting these epigenetic alterations.

*SSTR4*, a member of the somatostatin receptor family, has implications in various neurological and psychiatric conditions, encompassing cognitive function [9], AUD [10], and schizophrenia [11]. However, there is a scarcity of direct investigations centered on *SSTR4*. Our previous studies [12] validated that promoter-specific methylation of *SSTR4* contributes to the development of AUD and serves as a potential biomarker for assessing AUD severity by predicting chronic alcohol consumption. Nevertheless, the clinical significance of *SSTR4* in individuals with schizophrenia remains uncertain and warrants exploration. Consequently, this study seeks to investigate the role of *SSTR4* methylation in patients with schizophrenia.

## Methods and materials

### Data source

All data used in this study are collected from the public databases including Genotype-Tissue Expression (GTEx) project (https://www.gtexportal.org/), Gene Expression Omnibus (GEO, https://www.ncbi.nlm.nih.gov/geo/), and EWAS Open Platform [13] (https://ngdc.cncb.ac.cn/ewas/) including EWAS Atlas (https://ngdc.cncb.ac.cn/ewas/atlas), EWAS Data Hub (https://ngdc.cncb.ac.cn/ewas/datahub/) and EWAS Toolkit (https://ngdc.cncb.ac.cn/ewas/toolkit). All genomic locations are given in GRCh37.

### Tissue expression analysis

We utilized GTEx to analyze the expression patterns of SSTR4 across various tissues. Gene expression levels in different tissues were assessed by the median transcript count.

### Tissue methylation analysis

We employed the EWAS Data Hub [13] to analyze the methylation status of *SSTR4* in various tissues. Methylation status of *SSTR4* was evaluated at distinct locations, namely, the promoter region and gene body region. We assessed methylation levels at different tissue-specific positions (promoter and gene body regions) by calculating the average beta value.

### Aging pace analysis

We employed Pearson correlation analysis to examine aging pace. We compared the relationship between chronological age and *SSTR4* methylation levels. Additionally, we used the Epigenetic Clock (https://dnamage.clockfoundation.org/) to calculate epigenetic age and assessed its correlation with *SSTR4* methylation levels.

### Brain-Blood correlation in methylation of *SSTR4*

The inter-tissue DNA methylation consistency of SSTR4 between brain tissues and whole blood was assessed using the Blood Brain DNA Methylation Comparison Tool (https://epigenetics.essex.ac.uk/bloodbrain/). We compared the consistency in a total of 10 CpG sites from *SSTR4* including cg01277511, cg01471923, cg07032439, cg07676859, cg10019507, cg14631053, cg16502866, cg17586860, cg18197392, and cg22534145.

### Methylation quantitative trait loci analysis

We conducted an analysis of methylation quantitative trait loci (meQTLs) for CpG sites originating from *SSTR4* using the MeQTL EPIC Database (https://epicmeqtl.kcl.ac.uk/) [14] and mQTLdb (http://www.mqtldb.org/) [15]. The genetic variant that acts as meQTL will be further clumped due to the linkage disequilibrium (LD, *R*^2^ or D`> 0.2).

### LD trait analysis

To confirm what trait the meQTL may involve, LD trait analysis was conducted on LDlink Platform (https://ldlink.nih.gov/). We use the clumped meQTLs to examine the genetic variant affected trait from the summary of genome-wide association study.

### Ethics and approval

This study was approved by the Ethics Committee of The First Affiliated Hospital and College of Clinical Medicine of Henan University of Science and Technology (No. 2023–465), and written informed consent was obtained from each participant. All methods were carried out in accordance with relevant institutional guidelines and regulations.

## Results

### Abundant expression of *SSTR4* in brain tissues as well as whole blood

**Fig 1:**
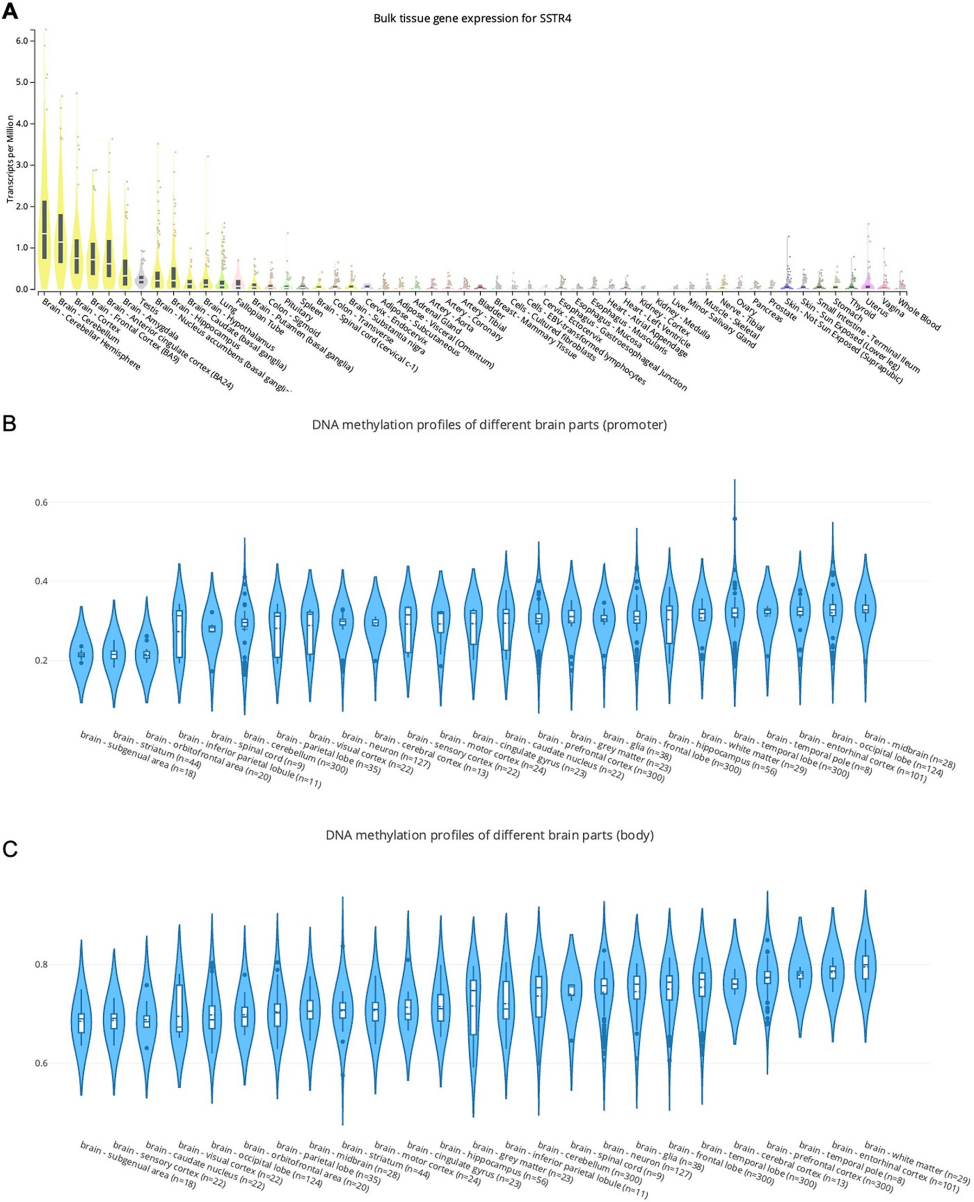
Expression and methylation status of *SSTR4* in different tissues. This plot illustrates the expression and methylation status of *SSTR4* in different tissues. **(A)** The top 10 *SSTR4*-expressed tissues are cerebellum hemisphere, cerebellum, cortex, frontal cortex, anterior cingulate cortex, amygdala, testis, nucleus accumbens, hippocampus, and caudate, respectively. **(B)** The methylation in promoter region of *SSTR4* is hypomethylated whereas **(C)** in gene body region of *SSTR4* is hypermethylated.

*SSTR4* (Somatostatin receptor 4) is implicated in an array of neuroendocrine, neurophysiological, and cognitive processes. Our previous finding indicated its abnormal methylation associated with alcohol dependence, one of substance use disorder that contributes to the risk of psychosis including schizophrenia. Encoded by the *SSTR4* gene, it is predominantly expressed in neural tissues, as evidenced by Genotype-Tissue Expression (GTEx) project data (Fig 1A). High expression levels are recorded in various brain regions, notably in the cerebellar hemisphere, cerebellum, cortex, frontal cortex (BA9), anterior cingulate cortex (BA24), amygdala, nucleus accumbens, hippocampus, caudate, and hypothalamus.

Further examination of the epigenetic landscape in EWAS Open Platform shows differential methylation patterns across the *SSTR4* gene locus, with promoter hypomethylation (mean beta-value < 0.5, Fig 1B) suggesting transcriptional activation and gene body hypermethylation (mean beta-value > 0.5, Fig 1C) potentially enhancing gene expression. These findings align with the GTEx expression data, suggesting that epigenetic modifications in the *SSTR4* gene are associated with its transcriptional activity and may influence its functional diversity in the brain.

It is important to note that no discernible differences were observed in the expression and methylation patterns of *SSTR4* between males (*n* = 349) and females (*n* = 186) diagnosed with schizophrenia, as demonstrated in S1 and S2 Figs in S1 Appendix.

### Methylation status of *SSTR4* associated with the pace of epigenetic aging condition

**Fig 2:**
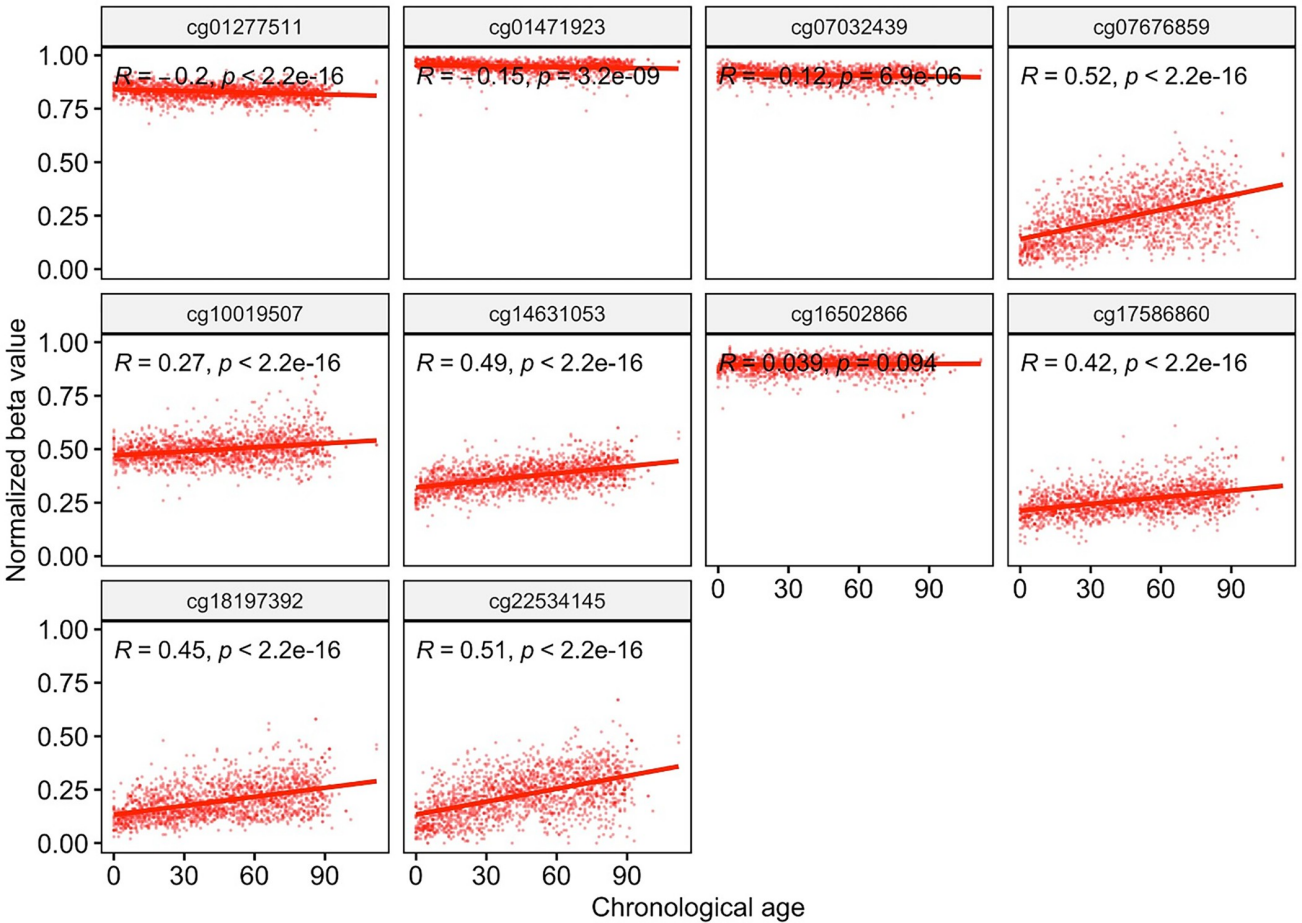
Correlation between methylation level of SSTR4 and chronological age. We conducted a comparison to assess the correlation between the normalized methylation levels of CpG sites within SSTR4 and chronological age. The significance of the correlation was determined using the Pearson correlation coefficient. Notably, the normalized beta values from CpG sites of SSTR4, with the exception of cg16502866, exhibited a significant correlation with chronological age.

We utilized epigenetic data from healthy individuals (*n*_male_ = 976, *n*_female_ = 822, age ranging from 0 to 112 yrs) sourced from the EWAS Open Platform to explore the association between the methylation status of *SSTR4* and aging conditions. A positive correlation (Fig 2) between chronological age and normalized beta values across multiple sites of *SSTR4* in healthy people. Correlation coefficients (R) ranged from 0.2 to 0.52, with all p-values being < 2.2e-16, denoting strong statistical significance.

### Altered and tissue-specific DNA methylation of *SSTR4* observed between healthy control and patients with schizophrenia

**Fig 3:**
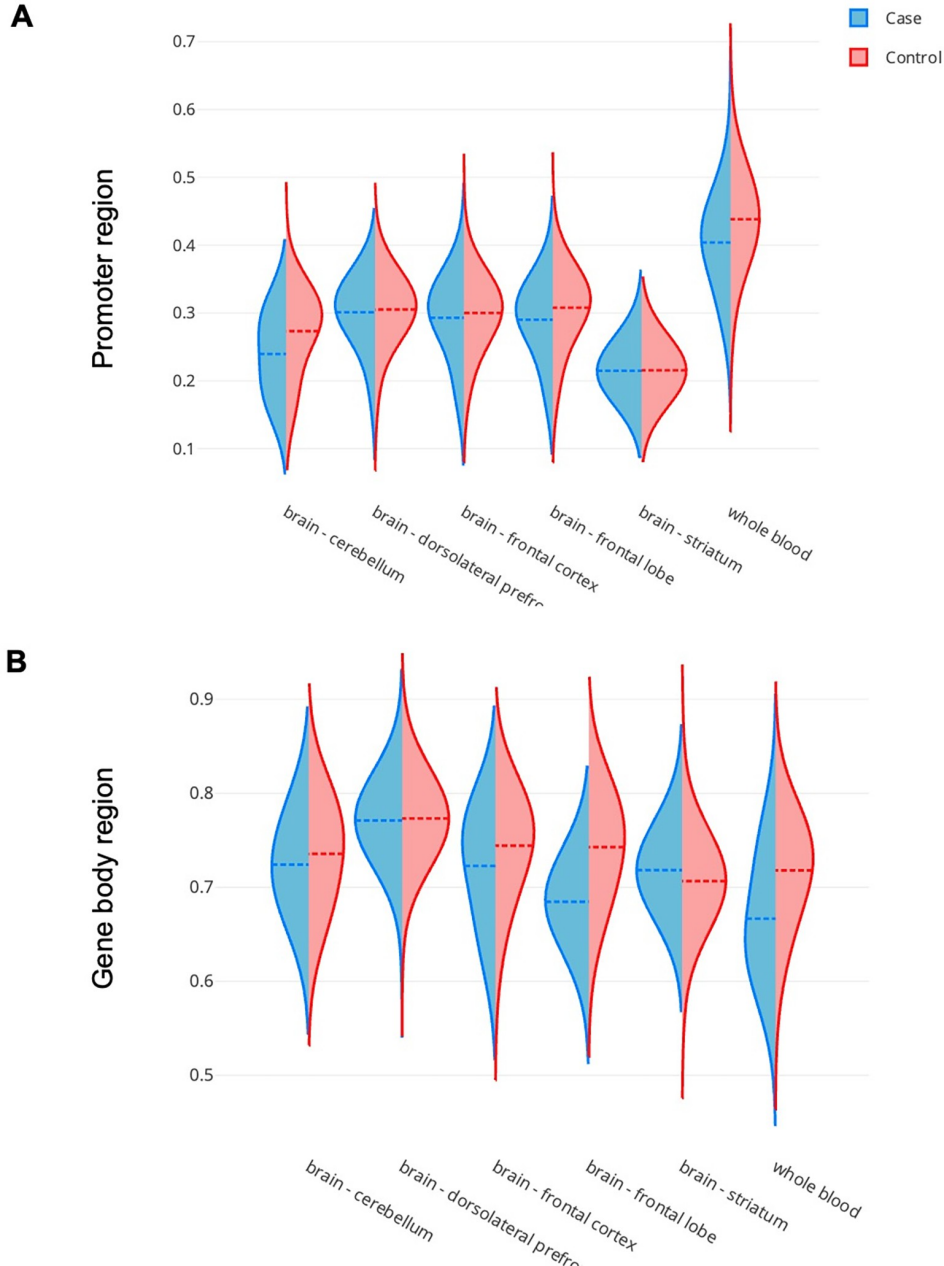
Contrasting methylation profiles of *SSTR4* in patients with schizophrenia versus healthy controls. In promoter region of *SSTR4* **(A)**, the methylation is obvious divergent in cerebellum and whole blood between patients with schizophrenia and healthy control. **(B)** In gene body region of *SSTR4*, except the dorsolateral prefrontal cortex, tissues including cerebellum, frontal cortex, frontal lobe, striatum, and whole blood showed the obvious divergent methylation between patients with schizophrenia and healthy controls.

The methylation status of *SSTR4* exhibits significant differences between healthy controls (*n*_control_ = 1,798) and patients with schizophrenia (*n*_case_ = 536) according to EWAS Open Platform, elucidating potential epigenetic mechanisms underlying the disease. In the promoter region (Fig 3A), a divergent methylation pattern is evident in tissues such as the cerebellum, frontal lobe, and whole blood, indicating differential regulation of *SSTR4* expression in these areas. This variation extends to the gene body region (Fig 3B), where notable disparities in methylation patterns are observed between controls and schizophrenia patients in the cerebellum, dorsolateral prefrontal cortex, frontal cortex, frontal lobe, and striatum, in addition to whole blood. These epigenetic alterations suggest that *SSTR4* may play a role in the pathophysiology of schizophrenia, possibly through its impact on gene expression and consequent neural functions.

### Genetic-epigenetic interaction in *SSTR4* associated with aging as well as cognitive function

Subsequently, we conducted an investigation to determine if the methylation of *SSTR4* was influenced by genetic variants, specifically methylation quantitative trait loci (meQTLs). Our findings reveal that distinct genetic variants were responsible for regulating the methylation levels of *SSTR4* at cg22534145, cg14631053, cg18197392, cg01277511, cg16502866, cg07676859, and cg17586860, each at different time points (see Table 1).

**Table 1:** Methylation quantitative trait loci of *SSTR4*.

Timepoint	SNP	SNP Chr	SNP Pos	A1	A2	MAF	CpG	CpG Chr	CpG Pos	beta	t-stat	Effect Size	p-value
Pregnancy	rs705935	6	158811727	C	T	0.301	cg22534145	20	23015936	-0.24927	-5.44223	0.01229	7.10E-08
Pregnancy	rs17691954	21	28644960	G	T	0.064	cg14631053	20	23015899	0.49315	5.98534	0.01983	3.32E-09
Birth	rs11464356	3	98819665	I	R	0.195	cg14631053	20	23015899	-0.2298	-5.57057	0.0139	3.51E-08
Birth	rs57499832	20	60759029	T	C	0.156	cg22534145	20	23015936	0.32522	5.44163	0.00042	7.10E-08
Childhood	rs201254961	5	11497624	D	R	0.131	cg22534145	20	23015936	-0.33649	-5.37801	0.01124	9.79E-08
Childhood	rs145337339	6	168835300	G	A	0.024	cg18197392	20	23015908	0.7312	5.52376	0	4.44E-08
Childhood	rs75235955	6	168873403	C	T	0.024	cg18197392	20	23015908	0.7312	5.52376	0	4.44E-08
Childhood	rs146757928	12	127125822	A	T	0.025	cg01277511	20	23016753	-0.75482	-5.66503	0.03096	2.03E-08
Adolescence	rs186364728	1	150495367	G	A	0.095	cg16502866	20	23015624	-0.37649	-5.70668	0.04149	1.60E-08
Adolescence	rs3109190	4	130799598	T	G	0.331	cg14631053	20	23015899	-0.20343	-5.59516	0.00968	2.99E-08
Adolescence	rs145240752	5	44061867	T	C	0.032	cg16502866	20	23015624	-0.62967	-5.52355	0	4.44E-08
Adolescence	rs145879288	9	77272444	T	C	0.019	cg14631053	20	23015899	0.71154	5.61635	0	2.66E-08
Middle Age	rs35367249	1	17879544	A	G	0.025	cg07676859	20	23015932	0.71461	5.49232	0.11496	5.46E-08
Middle Age	rs1034940	10	117437676	A	G	0.262	cg17586860	20	23015906	-0.28262	-5.44626	0.00238	7.01E-08

Moreover, our analysis of linkage disequilibrium (see Table 2) indicates that one of these meQTLs, rs705935, which affects cg22534145, is associated with cognitive function in genome-wide association studies. Additionally, our epigenome-wide association analysis (see Table 3) demonstrates that cg07676859 (regulated by rs35367249) and cg14631053 (regulated by rs17691954, rs11464356, rs3109190, and rs145879288) are associated with aging, while cg10019507, cg22534145, cg07676859, and cg18197392 are associated with substance use disorder.

**Table 2:** GWAS trait of meQTLs of *SSTR4*.

SNP	GWAS Trait	PMID	Alleles	R2	D’	P-value
rs705935	Educational attainment	35361970	A = 0.639, G = 0.361	0.68	0.85	5.00E-17
rs3109190	Body mass index	30239722	A = 0.687, T = 0.313	0.29	0.58	1.00E-14
rs3109190	Body mass index	30239722	C = 0.376, T = 0.624	0.23	0.52	2.00E-14
rs57499832	Liver enzyme levels (alkaline phosphatase)	33972514	C = 0.861, T = 0.139	0.27	0.60	3.00E-13
rs705935	Lung function (FVC)	30595370	C = 0.459, G = 0.541	0.29	0.64	4.00E-13
rs3109190	Body mass index	31669095	A = 0.687, T = 0.313	0.29	0.58	4.00E-12
rs57499832	Serum alkaline phosphatase levels	34594039	C = 0.858, T = 0.142	0.27	0.59	4.00E-12
rs3109190	Height	36224396	A = 0.359, C = 0.641	0.26	0.95	7.00E-11
rs705935	Heel bone mineral density	30048462	- = 0.532, A = 0.468	0.28	0.73	9.00E-11
rs3109190	Chronotype	30696823	C = 0.154, T = 0.846	0.29	0.91	1.00E-10
rs705935	Lung function (forced vital capacity)	36914875	C = 0.462, T = 0.538	0.28	0.63	3.00E-10
rs705935	Height (standard GWA)	37106081	C = 0.462, T = 0.538	0.28	0.63	6.00E-10
rs57499832	Serum alkaline phosphatase levels	33547301	A = 0.144, G = 0.856	0.26	0.58	2.00E-09
rs705935	Cognitive performance (MTAG)	30038396	C = 0.773, G = 0.227	0.26	0.73	1.00E-08
rs705935	Hemoglobin A1c levels	34594039	G = 0.514, T = 0.486	0.27	0.65	1.00E-08
rs3109190	Menarche (age at onset)	30595370	A = 0.269, G = 0.731	0.36	0.71	7.00E-08
rs705935	White blood cell count	30595370	C = 0.462, T = 0.538	0.28	0.63	1.00E-07
rs3109190	Body mass index	25673413	C = 0.574, T = 0.426	0.70	1.00	3.00E-07
rs705935	Height	36224396	C = 0.532, T = 0.468	0.52	0.99	9.00E-07
rs1034940	Hospital contact for infections	31712607	A = 0.746, G = 0.254	0.70	1.00	1.00E-06
rs3109190	Body mass index	25673413	C = 0.574, T = 0.426	0.70	1.00	1.00E-06
rs3109190	Body mass index	25673413	C = 0.574, T = 0.426	0.70	1.00	1.00E-06
rs3109190	Waist circumference	31513605	A = 0.708, T = 0.292	0.21	1.00	9.00E-06

**Table 3:** Epigenome-wide association of *SSTR4*.

CpG Site	Study PMID	Rank in study	P value	Trait	Case (Beta)	Control (Beta)	Effect size
cg07676859	28938548	56/1585	2.42E-31	prostate cancer	primary PCa	adjacent samples	-0.37
cg07676859	30866861	318/368	1.91E-33	breast cancer	breast cancer patients	healthy controls	0.41
cg07676859	33276343	323/1000	4.00E-165	aging	age		-
cg07676859	31552803	1640/4496	6.00E-10	smoking	smoking status		-
cg07676859	28890207	2419/14091	4.88E-04	oral squamous cell carcinoma (OSCC)	oral cancer (0.631389)	matched normal (0.316036)	0.32
cg07676859	30183087	2901/8245	4.37E-11	colorectal laterally spreading tumor	colorectal laterally spreading tumor (0.836)	normal control (0.621176)	0.21
cg07676859	29740534	4501/6167	9.13E-17	prostate cancer	prostate cancer	adjacent normal tissues	-
cg22534145	28938548	64/1585	1.20E-30	prostate cancer	primary PCa	adjacent samples	-0.29
cg22534145	26183421	1706/2209	6.48E-04	primary Sjögren’s Syndrome (pSS)	primary Sjögren’s syndrome	healthy controls	-
cg22534145	29740534	4743/6167	2.04E-16	prostate cancer	prostate cancer	adjacent normal tissues	-
cg22534145	28890207	6493/14091	4.88E-04	oral squamous cell carcinoma (OSCC)	oral cancer (0.56478)	matched normal (0.315242)	0.25
cg22534145	27651444	6951/9159	2.60E-05	smoking	current smokers	never smokers	0.01
cg18197392	28938548	1223/1585	3.02E-13	prostate cancer	primary PCa	adjacent samples	-0.22
cg18197392	30183087	1960/8245	7.06E-12	colorectal laterally spreading tumor	colorectal laterally spreading tumor (0.82)	normal control (0.478824)	0.34
cg18197392	31552803	3981/4496	9.40E-08	smoking	smoking status		-
cg18197392	28890207	12126/14091	9.77E-04	oral squamous cell carcinoma (OSCC)	oral cancer (0.580949)	matched normal (0.272076)	0.31
cg17586860	30036399	540/4974	3.35E-06	ic encephalomyelitis/chronic fatigue syndrome	ME/CFS patients (0.181067)	healthy controls (0.259386)	-0.08
cg17586860	28938548	903/1585	1.90E-15	prostate cancer	primary PCa	adjacent samples	-0.20
cg01471923	32496131	1/10	1.47E-10	polybrominated biphenyl exposure of male	polybrominated biphenyl exposure of male	matched males	-
cg01471923	30676242	148/1719	2.93E-06	polybrominated biphenyl exposure	total PBB levels		-
cg14631053	33276343	957/1000	7.74E-111	aging	age		-
cg14631053	30183087	1533/8245	2.02E-12	colorectal laterally spreading tumor	colorectal laterally spreading tumor (0.817)	normal control (0.527647)	0.29
cg16502866	29540343	654/800	8.50E-04	bladder cancer	pre-diagnostic bladder cancer cases	healthy controls	-
cg07032439	30183087	3894/8647	2.41E-17	adenoma	adenoma cases (0.58119)	normal control (0.851177)	-0.27

## Discussion

The present study elucidates the pivotal role of SSTR4 in neuroendocrine, neurophysiological, and cognitive processes through its abundant expression in various brain regions and whole blood. The genotype-tissue expression (GTEx) data substantiates the significant presence of SSTR4 in brain tissues, particularly within regions integral to cognitive and emotional processing, such as the cerebellum, cortex, and amygdala. This finding is in accordance with previous studies characterizing *SSTR4* expression patterns in the brain [16], which have highlighted its involvement in aging pathways and potential as a novel cognitive drug target.

The diverse methylation patterns observed across the *SSTR4* gene locus suggest complex epigenetic regulation, potentially contributing to the receptor’s functional diversity in the brain [12,17]. Promoter hypomethylation, indicating transcriptional activation, and gene body hypermethylation, potentially enhancing gene expression, align with GTEx expression data, suggesting a nuanced interplay between epigenetic modifications and transcriptional activity [12,18]. Our findings reveal dynamic allelic regulation of *SSTR4* DNA methylation over time, with various loci exhibiting allele-specific methylation changes from pregnancy to middle age, implying varying sensitivity to alcohol exposure [19–22]. The meQTLs of *SSTR4* that we have reported and cross-validated from public databases are trans-meQTLs. While cis-meQTLs, typically found near the methylation site they influence, are more numerous and play a significant role in gene expression regulation [23], trans-meQTLs, although fewer in number, exert a broader influence by affecting methylation at distant sites. They are particularly crucial for understanding responses to environmental changes [24] and complex disease mechanisms such as schizophrenia [25].

Patients with schizophrenia have exhibited a divergent epigenetic development trajectory [26]. This divergence prompts further exploration of the relationship between the methylation status of SSTR4 and the rate of epigenetic aging. Notably, the strong statistical significance observed in the correlation between chronological age and methylation at various *SSTR4* sites aligns with the growing body of research linking epigenetic markers to the aging process [27,28]. Furthermore, the genetic-epigenetic interactions, specifically the associations of certain meQTLs (such as rs705935) with cognitive function, a core symptom domain in schizophrenia patients with implications for functional and social outcomes [29], and aging (rs17691954, rs11464356, rs3109190, and rs145879288) [30], underscore the intricate genetic underpinnings shaping *SSTR4*’s epigenetic landscape. It is noteworthy that the connection between meQTLs and cognitive abilities offers a genetic perspective on the susceptibility of abnormal SSTR4 methylation to psychostimulant exposure [12]. This association sheds light on the potential role of *SSTR4* in neurocognitive disorders and warrants further investigation.

Somatostatin itself has been reported to exert inhibitory effects on pyramidal neurons, resulting in hypofrontality, a phenomenon associated with psychiatric disorders such as schizophrenia and addiction [31]. The observed differential DNA methylation of *SSTR4* in specific tissues among healthy controls and individuals with schizophrenia provides valuable insights into potential epigenetic mechanisms associated with psychiatric disorders. The variation in methylation patterns in crucial brain regions and blood samples suggests the potential utility of *SSTR4* methylation status as a peripheral biomarker for assessing both aging and cognitive impairment.

The potential impact of antipsychotics or smoking on the methylation status of *SSTR4* gene remains a complex issue requiring further investigation. While specific studies on *SSTR4* methylation in the context of antipsychotic use or smoking are limited, broader research suggests that antipsychotic drugs can induce changes in DNA methylation patterns, potentially influencing gene expression and contributing to therapeutic effects, side effects, and pharmacological actions at the cellular level [32–34]. Additionally, smoking is known to have widespread effects on DNA methylation across the genome, which may interact with the pharmacodynamics of antipsychotic drugs and influence neurotransmission-related gene expression. The impact of antipsychotics or smoking on *SSTR4* methylation likely represents a complex interplay between genetic, environmental, and pharmacological factors, warranting further research to elucidate the specific epigenetic mechanisms involved and their implications for psychiatric disorders and treatments.

Research investigations into the relationship between the methylation status of the SSTR4 gene and cognitive function have yielded insights into potential epigenetic influences on cognition and disease states. While Grosser et al. [34] found no significant difference in SSTR4 promoter methylation between Alzheimer’s disease patients and controls, other studies have suggested associations between DNA methylation and cognitive impairment. Grove et al. [35] reported a link between oxytocin receptor methylation and general cognition in psychotic disorders, and our previous study [12] observed lower SSTR4 methylation levels in individuals with alcohol dependence. Furthermore, Liu et al. [36] discussed the potential involvement of DNA methylation in neuronal memory coding and age-related cognitive decline. These findings collectively highlight the complex interplay between epigenetic mechanisms, such as SSTR4 methylation, and cognitive processes, underscoring the need for further research to fully elucidate the implications of epigenetic regulation in cognitive function and pathology.

While the direct relationship between methylation of SSTR4 and schizophrenia has not been explicitly studied, a substantial body of research has implicated aberrant DNA methylation patterns in the pathogenesis of this psychiatric disorder. Studies have reported hypomethylation of genes like HTR2A [37], dysregulation of DNA methylation machinery enzymes [38], increased methylation of the 5HTR1A promoter [39], and genome-wide differentially methylated regions [40] in individuals with schizophrenia. Although the specific role of SSTR4 methylation remains unexplored, these findings collectively underscore the significance of epigenetic mechanisms, particularly DNA methylation, in schizophrenia’s etiopathology, suggesting that altered methylation patterns could represent a common feature across various genes and pathways implicated in this complex neuropsychiatric disorder.

The identification of reliable biomarkers that can accurately assess cognitive function in individuals with schizophrenia has the potential to revolutionize clinical management. SSTR4 is a promising candidate biomarker, as our study implicated its role in modulating cognitive processes and demonstrated alterations in SSTR4 expression and function in schizophrenia patients, correlating with cognitive deficits. Incorporating SSTR4 as a biomarker could facilitate early detection, disease monitoring, and evaluation of cognitive-enhancing therapies, enabling personalized treatment approaches and potentially improving long-term outcomes for individuals with schizophrenia.

In conclusion, the present study supports the hypothesis that *SSTR4* is not only crucial for various brain functions but is also intricately regulated at an epigenetic level, influenced by both aging and genetic factors. These results lay the groundwork for future research into the therapeutic potential of targeting *SSTR4* in neurodegenerative and psychiatric conditions. Further exploration of the epigenetic regulation of *SSTR4* may illuminate new strategies for modulating its activity in disease states.

## Supporting information

S1 File (DOCX)
