# Supplementary material for: Allele-specific methylation of SSTR4 associated with aging and cognitive functions in patients with schizophrenia
Source: PLoS One. 2025 Feb 5;20(2):e0303038. doi: 10.1371/journal.pone.0303038 (PMC11798447; doi:10.1371/journal.pone.0303038)
Supplement: S1 File — (DOCX) [file pone.0303038.s001.docx]

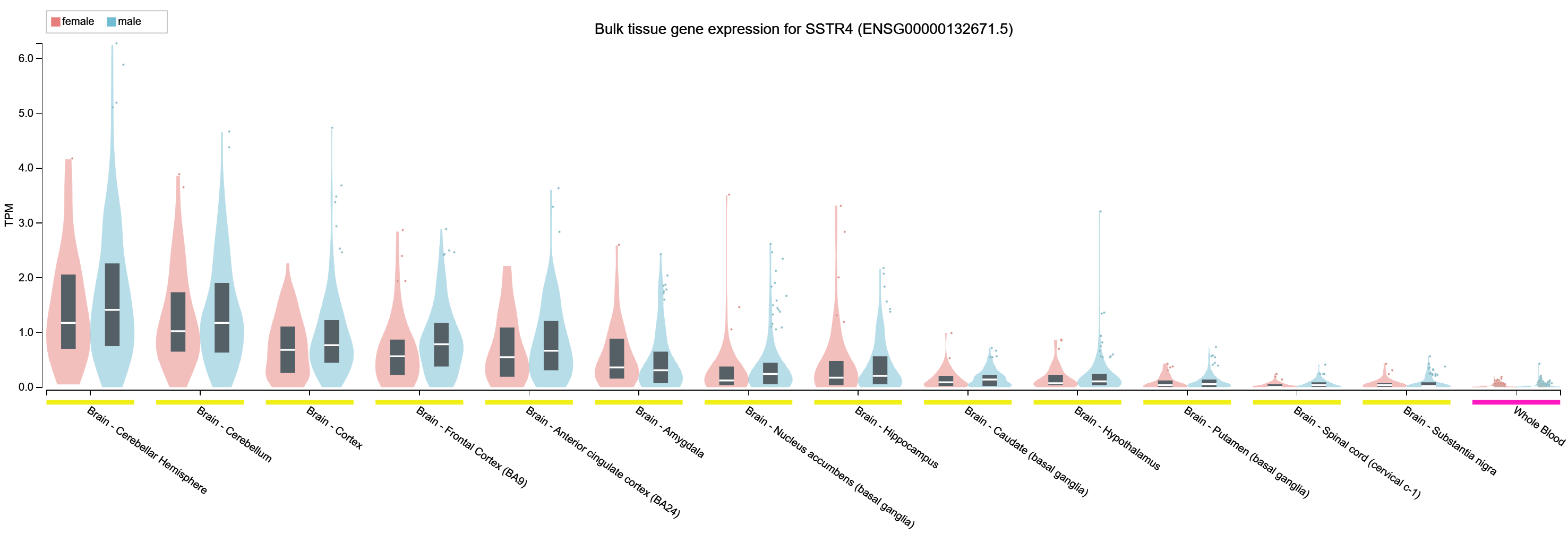


Supplementary Fig S 1 SSTR4 expression between male and female in different tissues.

The expression levels of SSTR4 exhibited comparable patterns across tissues in both male and female subjects.


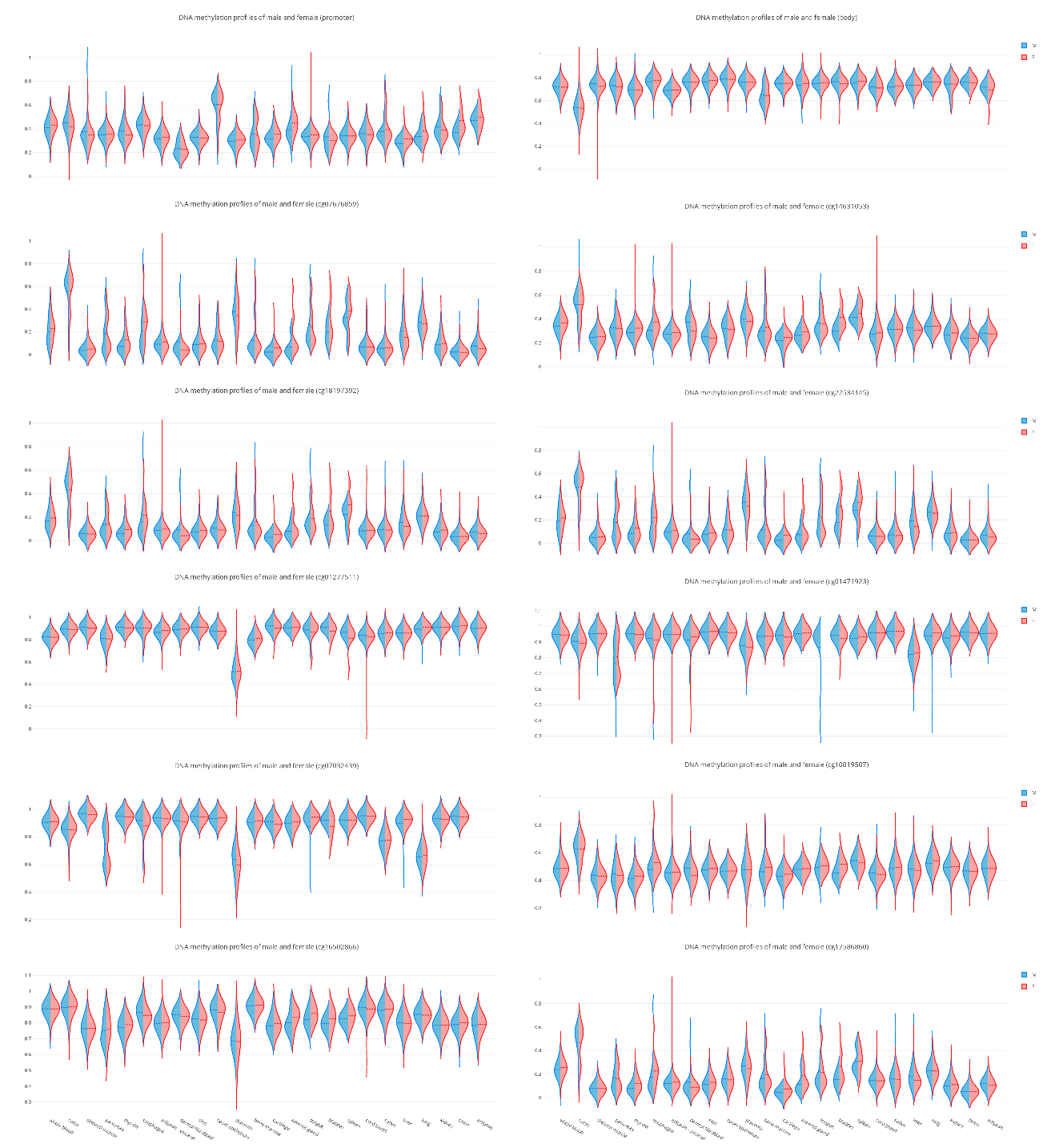


Supplementary Fig S 2 Methylation level of SSTR4 between males and females

The presented plot depicts the methylation profiles of SSTR4 across various tissues in male and female cohorts. In each tissue, the CpG sites within SSTR4 exhibited consistent methylation levels between males and females.
